# The role of the ‘obligation to help others’ within the superwoman schema: implications for physical activity in African American women with low activity levels

**DOI:** 10.1007/s10865-026-00678-y

**Published:** 2026-05-24

**Authors:** Allison M. Sweeney, Lisa Brockington, Timothy Simmons, Dawn K. Wilson, Pamela Martin, Nicole Zarrett

**Affiliations:** 1 Department of Biobehavioral Health and Nursing Science, College of Nursing, University of South Carolina, 1601 Greene St, Columbia, SC 29208 USA; 2 Department of Psychology, University of Maryland, 4094 Campus Dr, College Park, MA 20742 USA; 3 Department of Psychology, University of South Carolina, 1512 Pendleton St, Columbia, SC 29208 USA; 4 Department of African American Studies, University of South Carolina, 817 Henderson St, Columbia, SC 29208 USA

**Keywords:** Physical activity, African American women, Superwoman schema, Accelerometry

## Abstract

The Superwoman Schema (SWS) provides a critical framework for understanding how African American (AA) women respond to stress resulting from adversity. While past studies have found that components of the SWS are associated with negative mental health outcomes, little research has evaluated physical activity (PA) and has been limited by self-reports. This study examined whether the ‘obligation to help others’ subscale of the SWS was associated with PA after accounting for selected social-motivational variables commonly examined in PA research, including self-efficacy, self-determined motivation, and social support from family. Baseline data from 301 AA women (*M*BMI = 35.89 ± 7.60; *M*age = 53.47 ± 13.66) who participated in the ‘Together Everyone Achieves More Physical Activity’ randomized controlled trial were included. Participants completed a baseline survey, including the ‘obligation to help others’ subscale of the SWS and measures of self-efficacy, self-determined motivation, and social support. Participants wore an ActiGraph accelerometer for 7-days to assess daily minutes of light and moderate to vigorous PA (MVPA). Linear regression models revealed that the ‘obligation to help others’ was positively associated with greater light PA (*B* = 3.90, *SE* = 1.88, *p* = 0.039), which is contrary to past research on the SWS with self-reported PA. Only autonomous motivation was significantly associated with greater light (*B* = 6.91, *SE* = 1.98, *p* = 0.001) and MVPA (*B* = 0.27, *SE* = 0.08, *p* = 0.001). This study highlights the importance of addressing barriers and sources of resiliency related to the SWS, including the obligation to help others, in future PA interventions for AA women.

*Trials registration* ClinicalTrials.gov NCT (# NCT05519696).

## Introduction

Although there have been significant improvements in life expectancy over the last decade, large health disparities remain, especially among African American women (Cunningham et al., 2017). African American women experience disproportionately high rates of cardiovascular disease beginning in early adulthood, along with earlier onset of chronic health conditions shaped by structural inequities such as limited access to preventive care, chronic stress exposure, socioeconomic constraints, and local environmental factors that influence daily stress and access to care (Cunningham et al., 2017; Simons et al., 2021; Tsao et al., 2022). While physical activity (PA) is well-established as a critical protective factor for reducing risk for cardiovascular disease and all-cause mortality (Lee et al. 2012a; Sheehy et al. 2020), African American women face compounding social and structural barriers that interfere with regular PA such as caregiver responsibilities, lack of access, and safety concerns (Joseph et al., 2015; Mathew Joseph et al., 2018; Obi et al., 2022; Siddiqi et al., 2011).

One framework for understanding the relationship between barriers, lived experiences, and health behaviors among African American women is the Superwoman Schema (SWS) conceptual framework. Developed by Woods-Giscombé (2010), this framework was derived from a series of focus groups with a socioeconomically diverse group of African American women, who shared their interpretations of the “superwoman role” based on their lived experiences (Woods-Giscombé, 2010). The qualitative analysis revealed the adoption of a cultural schema that emphasizes five key characteristics: (1) obligation to show strength, which involves projecting a persona that remains assertive and independent when facing challenges; (2) suppression of emotions, which entails internalizing feelings rather than expressing a wide range of emotions externally, and is often interpreted as reinforcing the notion of being “strong”; (3) resistance to vulnerability, characterized by projecting independence and self-reliance, along with a reluctance to seek assistance from others; (4) drive to succeed despite limited resources, reflecting a deeply ingrained intrinsic motivation to achieve despite challenges and a lack of support; and (5) obligation to care for others, which involves selflessly helping those around them, often at the expense of one’s own well-being.

The SWS is theorized to have developed as a coping response to confront racial- and sex-based adversity, and is critical for understanding the intrapersonal, interpersonal, and socio-cultural factors that shape African American women’s health and well-being (Nelson et al., 2016; Parks & Hayman, 2024; Sheffield-Abdullah & Woods-Giscombe, 2021; Woods-Giscombé, 2010; Woods-Giscombe et al., 2019). Numerous researchers have proposed that the SWS can be conceptualized as a ‘double-edged sword,’ highlighting the importance of considering both the context and different health-related outcomes to understand adaptive and maladaptive tendencies (Abrams et al., 2014; Watson-Singleton et al., 2024; Woods-Giscombe et al., 2019). Specifically, while some studies have found that certain aspects of the SWS may serve as adaptive factors among women with lower socioeconomic status (Erving et al., 2024) and for coping with racial discrimination (Allen et al., 2019), numerous studies provide convergent evidence of the maladaptive outcomes associated with the SWS, including greater depression, anxiety, and stress (Castelin & White, 2022; Jones et al., 2021; Knighton et al., 2022; Leath et al., 2022).

Greater endorsement of the SWS has also been associated with worse physical health outcomes, including worse quality sleep, unhealthy eating, and hypertension (Farr et al., 2025; Mclaurin-Jones et al., 2021; Perez et al., 2023; Woods-Giscombe et al., 2019). To the authors’ knowledge, only two previous studies have evaluated the relationship between the SWS and PA (Farr et al., 2025; Woods-Giscombe et al., 2019). Specifically, Farr et al. (2025) found that only the emotional suppression facet of the SWS was negatively associated with PA, whereas Woods-Giscombé et al. (2019) found that all aspects of the SWS were negatively associated with PA, with the obligation to help others subscale having the largest correlation. Notably, both of these studies included self-reported PA, which may be a limitation, given that self-reports of PA are prone to overestimation and tend to have small to moderate correlations with objective device-based measures (e.g., accelerometers) (Gorzelitz et al., 2018; Prince et al., 2008). Furthermore, these past studies have not differentiated between PA intensities (e.g., light and moderate to vigorous activities), which may be important to consider given that light PA (LPA) is also associated with important health benefits, including cardio-metabolic health, ageing, and weight status (Bourdier et al., 2023; Chastin et al., 2019; Hamm et al., 2025).

In the present study, we propose evaluating the extent to which the ‘obligation to help others’ component of the SWS adds to existing theory-based constructs (e.g., self-efficacy, social support, self-determined motivation) to further enhance understanding of PA engagement among African American women. The present study focuses specifically on the ‘obligation to help others’ component of the SWS, as this component demonstrated the largest correlation with PA in the existing literature at the time of study design (Woods-Giscombe et al., 2019). One recent study also found that the ‘obligation to help others’ component of the SWS was significantly associated with lower self-rated health, while all other components of the SWS were not significant predictors (Erving et al., 2024). Consistent with past research that has found that caregiving is often viewed as a key barrier to PA among African American women (Joseph et al., 2015; Mathew Joseph et al., 2018; Obi et al., 2022; Siddiqi et al., 2011), we propose that the ‘obligation to help others’ component of the SWS taps into aspects of African American women’s lived experiences that may complement constructs from existing social-motivational theoretical frameworks.

Efforts to develop PA interventions for African American women have largely centered around a set of essential theoretical constructs from social-motivational theories of health behavior change, including self-efficacy, social support, and self-determined motivation. Prior reviews of PA interventions for African American women (Bland & Sharma, 2017; Jenkins et al., 2017; Nastvogel, 2025) have demonstrated that prominent intervention strategies include goal-setting, problem-solving, and self-monitoring, which are derived from Social Cognitive Theory, and theorized to be critical behavioral skills for promoting self-efficacy, or confidence in one’s ability to meet goals (Bandura, 2004). Past reviews have also demonstrated that social support is a frequently used intervention strategy (Bland & Sharma, 2017; Jenkins et al., 2017; Nastvogel, 2025), with numerous studies finding that social support from peers, family, and/or friends is associated with greater PA among African American women (Andersen et al., 2015; Coulon et al., 2013; Johnson et al., 2014; Sharma et al., 2005; Sweeney et al., 2025, 2026). Additionally, Self-Determination Theory (Ryan & Deci, 2000), has been applied extensively in the development of PA interventions, with numerous studies indicating that autonomous motivation (i.e., motivation from internally-driven factors, such as values, enjoyment) is associated with greater PA engagement (Fortier et al. 2012; Landry and Solmon 2004; Teixeira et al. 2012a, b). Thus, the purpose of the present study is to evaluate whether the ‘obligation to help others’ component of the SWS further enhances our understanding of PA engagement by accounting for unique variance when compared with existing social-motivational constructs.

Although there is extensive support for the importance of social-motivational constructs for understanding PA among African American women, there is also broad recognition of the importance of addressing the unique lived experiences and prominent cultural values, beliefs, and norms of African American women (Kong et al., 2014; Moore Hubbell et al., 2023; Obi et al., 2022). To the authors’ knowledge, no previous studies have directly compared the effect of the SWS (including the ‘obligation to help others’ component) for predicting health-related outcomes when simultaneously accounting for social-motivational constructs, or whether the ‘obligation to help others’ component relates to objectively-measured PA and different PA intensities. Specifically, the present study evaluated whether the ‘obligation to help others’ component of the SWS relates to accelerometry-assessed LPA and moderate to vigorous physical activity (MVPA) across a 7-day period among African American women. We hypothesized that greater endorsement of the ‘obligation to help others’ component of the SWS would relate to lower daily minutes of MVPA and LPA. Additionally, we hypothesized that the ‘obligation to help others’ component of the SWS would have a significant independent main effect on MVPA and LPA and account for unique variance after adjusting for self-efficacy, social support from family, and self-determined motivation for PA.

## Methods

### Participants

Baseline data from 301 African American women participating in the ongoing ‘Together Everyone Achieves More Physical Activity’ (TEAM-PA) randomized controlled trial were included in the present study (Sweeney et al., 2023). Participants were recruited in collaboration with local community partners in [Southeast U.S. – South Carolina]. Participants were eligible if they were 1) *≥* 18 years old; 2) self-identified as an African American or Black female; and 3) engaged in < 60 min of self-reported MVPA/week for the last three months. Exclusion criteria included: (1) having a cardiovascular or orthopedic condition that limits PA; (2) inability to walk without a walker/cane; (3) pregnancy; or (4) uncontrolled blood pressure (systolic > 180 mmHg/diastolic > 110 mmHg).

### Procedures

Participants completed a phone screening, including the short-version of the International Physical Activity Questionnaire (Craig et al., 2003) and the Physical Activity Readiness Questionnaire (National Academy of Sports Medicine, 2019). If participants reported a history of cardiovascular disease, high blood pressure, orthopedic issues, or other conditions that would limit PA, medical approval was required from a primary care provider. Eligible participants then completed a group orientation session to receive detailed information, complete a blood pressure assessment, and provide informed consent.

Participants then attended a 2-week run-in phase prior to randomization to group condition to collect baseline measures. All data collection and group sessions took place at local community recreation centers in collaboration with our community partners, which served as hubs for implementing the study. Participants were required to attend at least one run-in session to be included in the group randomization to assure commitment to being enrolled in the intervention or comparison program. Baseline assessments, which typically lasted one hour or less, included measures of height and weight and completion of a self-report survey. Participants also received a wrist-worn ActiGraph accelerometer and were asked to wear the device for 7 days to measure baseline levels of PA. Participants returned the accelerometer devices by the start of the group session in week 3 prior to revealing group randomization. Participants received $25 for completing baseline measures. The study was approved by the [University of South Carolina] Institutional Review Board.

### Measures

#### Demographic measures

Demographic measures were obtained during enrollment, including age, income, marital status, education, and the number of children living at home.

#### Body Mass Index (BMI)

Trained research staff took two measures of weight and height using a Seca digital scale and a Shorr height board, respectively, in a private room. An average height and an average weight were calculated using these two measurements and used to calculate BMI (kg/m^2^).

#### Superwoman schema—obligation to help others

The ‘obligation to help others’ subscale, drawn from the Giscombe Superwoman Schema Questionnaire, was used in the TEAM-PA trial (Woods-Giscombe et al., 2019). Nine items were scored on a 6-point scale (1 = very untrue of me, 6 = very true of me). An example item is, “There is no time for me because I am always taking care of others.” Prior research has demonstrated the construct validity of this scale by showing that it is positively associated with stress and depression (Woods-Giscombe et al., 2019), and distinct from other cultural constructs, such as John-Henryism (Perez et al., 2023). Past studies have found this subscale to have adequate internal consistency (α = 0.88) and test-retest reliability (*r* = 0.89) and have typically calculated a sum or an average for this subscale (Allen et al., 2019; Steed, 2014; Woods-Giscombe et al., 2019). In this study, this scale had adequate internal consistency (α = 0.90).

#### Self-efficacy for PA

Self-Efficacy was assessed using a modified version of a scale for exercise self-efficacy (Sallis et al., 1988). Thirteen items were scored on a 5-point scale (1 = not at all confident, 5 = extremely confident). Items assessed confidence in sticking with being physically active when dealing with potential barriers, such as “my family is demanding a lot of time from me” and “I feel tired”. Past research has demonstrated adequate internal consistency for this scale (α = 0.83–0.85) and have typically calculated this scale as a sum or an average (Sallis et al., 1988; Yeary et al., 2011). In the present study, this scale was found to have adequate internal consistency (α = 0.93).

#### Autonomous and controlled motivation for PA

Motivation for PA was measured using the Behavioral Regulation in Exercise Questionnaire (BREQ-3) (Mullan et al., 1997; Wilson et al., 2007). The BREQ-3 consists of subscales assessing controlled motivation (extrinsic motivation, introjected motivation) and autonomous motivation (identified motivation, integrated motivation, intrinsic motivation). In the TEAM-PA trial, the amotivation subscale was not used as we expected minimal variance on this subscale. Twenty items were scored on a 6-point scale (1 = very untrue of me, 6 = very true of me). Sample items included: “I feel guilty when I don’t exercise” and “I get satisfaction from participating in exercise”. Past research has demonstrated adequate internal consistency for all subscales (α = 0.70–0.93) and have typically calculated subscales as a sum or average (Landry & Solmon, 2004; Mullan et al., 1997). A controlled motivation summary score was created by averaging the values for the extrinsic and introjected subscales, and an autonomous motivation summary score was created by averaging the values for the identified, integrated, and intrinsic motivation subscales. In the present study, this scale was found to have adequate internal consistency (controlled motivation: α = 0.76; autonomous motivation: α = 0.93).

#### Social support for PA from family

Social support was measured using the Social Support for Exercise scale (Sallis et al., 1987). Seven items were scored on a 6-point scale (1 = never, 6 = very frequently). Items assessed whether family members provided different forms of social support for PA in the last two weeks, such as “were physically active with you” or “encouraged you to be physically active”. Past research has demonstrated adequate internal consistency for this scale (α = 0.84–0.91) and has typically calculated this scale as a sum or an average (Lee et al. 2012a, b; Sallis et al. 1987). Internal consistency for the scale was high in this study (α = 0.91).

#### Physical activity (LPA, MVPA)

Measurement of PA duration and intensity was obtained from wrist-worn ActiGraphs (wGT3X-BT), which recorded PA in 60-second epochs. Accelerometry data were downloaded with ActiLife software and saved in a raw format containing acceleration data. These files were summarized in R using the GGIR package (Migueles et al., 2019) using the Euclidean Norm Minus One metric with no imputation. Thresholds for LPA and MVPA were adapted from Hildebrand and colleagues (Hildebrand et al., 2014, 2017).

### Statistical analyses

Participants had an average of 6.04 days (*SD* = 1.49) of at least 10 h of wear/day. LPA and MVPA were reviewed for normality. Prior to analysis, outliers were identified and recoded to reflect a maximum of three times the interquartile range using a Winsorizing approach (Dixon, 1960), which resulted in recoding *≤* 2% of daily values. Additionally, a square root transformation was applied to MVPA to achieve a normal distribution. To calculate average daily minutes of MVPA and LPA across the 7-day wear period, mixed models were used which included a random effect for individuals and used variance weighting by the inverse of daily wear time proportions to predict PA outcomes. This approach results in those with a higher proportion of missing wear time being down weighted (vs. those with less missingness) and improves precision in estimating PA (Yue Xu et al., 2018). Individual coefficients were extracted from each model to calculate average daily minutes of MVPA and LPA, respectively. Scores for each scale, including self-efficacy, social support, autonomous motivation, controlled motivation, and the ‘obligation to help others’ component of the SWS, were calculated by norming each item before summation to allow each item to contribute equally to the overall score. Summed scale items were transformed to z scores to aid in interpretation.

A series of linear regression models were used to assess average daily minutes of LPA and MVPA. For each outcome, model 1 included demographics only (age, BMI, marital status, children living at home, and education), model 2 included the addition of the social-motivational variables, and model 3 added the ‘obligation to help others’ component of the SWS. Age and BMI were mean centered, and all other covariates were coded as dichotomous variables, including marital status (1 = married, 0 = unmarried), children living at home (1 = at least 1 child at home, 0 = no children at home), and education (1 = 4-year college education or greater, 0 = no college education).

Additionally, follow-up analyses examined the relationships between autonomous motivation, controlled motivation, the ‘obligation to help others’ subscale of the SWS, and PA outcomes. We computed separate scores for each of the sub-scales of the BREQ-3 (extrinsic, introjected, identified, integrated, and intrinsic motivation). Linear regressions predicting LPA and MVPA were conducted, with the final model including covariates (age, BMI, marital status, children living at home, and education), all motivation subtypes, and the ‘obligation to help others’ subscale of the SWS.

## Results

### Demographic data

Participants were between the ages of 18 and 80, with an average age of 53.47 ± 13.64 (see Table 1). Approximately 39.2% were married. Median annual household income was $55,000 to $69,999, and 57.48% had a college degree or greater. Most participants had a BMI in the obese range (≥ 30 kg/m^2^), with an average BMI of 35.89 ± 7.60. At baseline, approximately 53.8% of participants (*n* = 162) reported that they were currently taking blood pressure medication.

**Table 1:** Participant baseline characteristics (*N* = 301)

Variable	Value
Age (years)	53.47 (13.66)
Married	118 (39.2%)
Children living at home	103 (34.2%)
Education	
High school or less	35 (11.6%)
Some college	93 (30.9%)
College or higher	73 (24.3%)
Graduate Degree	100 (33.2%)
Income	
< $24,999	39 (12.9%)
$25,000–$39,999	51 (17.0%)
$40,000–$54,999	55 (18.3%)
$55,000–$69,999	58 (19.3%)
$70,000–$84,999	43 (14.3%)
$85,000 or more	51 (17.0%)
Not reported	4 (1.3%)
Body Mass Index (kg/m^2^)	35.89 (7.6)
Normal Weight (≤ 24.9 kg/m^2^)	17 (5.6%)
Overweight (25–29.9 kg/m^2^)	51 (16.9%)
Obese (> 30 kg/m^2^)	233 (77.4%)
Blood Pressure Medication	
Yes	162 (53.8%)
No	139 (46.2%)

Values are Mean ± SD or frequencies and percentages

### Correlation analyses

Table 2 presents means and bivariate correlations for the predictor, outcome, and demographic variables. LPA was positively correlated with autonomous motivation (*r* = 0.21), but not controlled motivation, self-efficacy, or the ‘obligation to help others’ component of the SWS. MVPA was negatively correlated with age only (*r* = − 0.28). Additionally, the ‘obligation to help others’ component of the SWS was positively associated with controlled motivation (*r* = 0.21) and BMI (*r* = 0.17) and negatively correlated with age (*r* = − 0.31).

**Table 2:** Bivariate correlations between physical activity outcomes, social-motivational factors and SWS-obligation to help others

	*M (SD*)	1	2	3	4	5	6	7	8	9
1. LPA	115.88 (30.35)	−								
2. MVPA (square-root transformed)	4.77 (1.23)	0.68**	−							
3. Self-Efficacy	3.58 (0.98)	− 0.06	0	−						
4. Controlled Motivation	2.7 (0.89)	0.06	− 0.01	0	−					
5. Autonomous Motivation	4.0 (1.1)	0.21**	0.14	0.22**	0.39**	−				
6. Social Support fom Family	2.43 (1.21)	0.1	0.06	0.12*	0.25**	0.33**	−			
7. SWS—Obligation to help others	3.71 (1.08)	0.06	0.07	0.01	0.21**	− 0.10	− 0.07	−		
8. BMI	35.89 (7.57)	− 0.21**	− 0.08	0.03	0	− 0.15**	− 0.02	0.17*	−	
9. Age	53.47 (13.66)	0.01	− 0.28**	0.11*	0.09	0.2**	0.06	− 0.31**	− 0.08	–

* *p* < 0.05; ** *p* < 0.01

LPA = light physical activity; MVPA = moderate to vigorous physical activity; SWS = Superwoman schema; BMI = body mass index

### Effects of the ‘obligation to help others’ and social-motivational factors on LPA and MVPA

As shown in Table 3, a hierarchical approach was used to examine the associations between self-efficacy for PA, autonomous motivation, controlled motivation, social support from family, and the ‘obligation to help others’ component of the SWS on daily minutes of LPA. The first step of the regression included demographics only, which was significant *F*(5, 295) = 5.54, *p* < 0.001) and explained 8.6% of the variance (*R*^2^= 0.086). The addition of the social-motivational variables in step 2 resulted in a significant change in the model, *F*_change_ (4, 291) = 3.33, *p* = 0.011, which explained an additional 4% of the variance (*R*^2^ = 0.126). At the final step of the regression, the ‘obligation to help others’ subscale of the SWS was entered, resulting in a significant change in the model, *F*_change_ (1, 290) = 4.29, *p* = 0.039, which explained an additional 1.3% of the variance (*R*^2^ = 0.139). In the final model, there was a significant positive main effect of the ‘obligation to help others’ component of the SWS, such that greater endorsement of the ‘obligation to help others’ component of the SWS was associated with greater daily minutes of LPA (*B* = 3.90, *SE* = 1.88, *p* = 0.039). There was also a significant positive main effect of autonomous motivation (*B* = 6.91, *SE* = 1.98, *p* = 0.001), such that higher autonomous motivation was associated with greater daily minutes of LPA. There were no significant main effects for self-efficacy, controlled motivation, or social support from family.

As shown in Table 4, the same hierarchical modeling approach was used to examine daily minutes of MVPA (square root transformed values). The first step of the regression included demographics only, which was significant (*F*(5, 295) = 6.16, *p* < 0.001) and explained 9.5% of the variance (*R*^2^= 0.095). The addition of the social-motivational variables in step 2 resulted in a significant change in the model, *F*_change_ (4, 291) = 3.15, *p* = 0.015, which explained an additional 3.7% of the variance (*R*^2^ = 0.132). However, the addition of the ‘obligation to help others’ subscale of the SWS in the final step did not significantly change the model, *F*_change_ (1, 290) = 0.04, *p* = 0.845. In the final model, there was no significant main effect of the ‘obligation to help others’ component of the SWS on MVPA. The only significant predictor was autonomous motivation (*B* = 0.27, *SE* = 0.08, *p* = 0.001), such that greater autonomous motivation was associated with greater daily minutes of MVPA. There were no significant main effects for self-efficacy, controlled motivation, or social support from family.

**Table 3:** Linear regression models predicting average daily minutes of LPA from demographics, social-motivational factors and SWS-obligation to help others

	Model 1				Model 2				Model 3			
	B	SE	*p*	95% CI	B	SE	*p*	95% CI	B	SE	*p*	95% CI
Intercept	120.19	3.31	< 0.001	113.67, 126.71	119.23	3.28	< 0.001	112.77, 125.69	119.66	3.27	< 0.001	113.22, 126.10
Age	− 0.07	0.15	0.666	− 0.36, 0.23	− 0.13	0.15	0.377	− 0.43, 0.16	− 0.07	0.15	0.634	− 0.38, 0.23
Children at home	-0.45	4.33	0.917	− 8.98, 8.08	− 0.97	4.28	0.821	− 9.38, 7.45	− 2.37	4.31	0.582	− 10.84, 6.10
Married	5.72	3.50	0.104	− 1.17, 12.61	6.36	3.46	0.067	− 0.44, 13.17	6.20	3.44	0.073	− 0.57, 12.96
Education	− 11.13	3.46	0.001	− 17.94, − 4.31	− 9.64	3.46	0.006	− 16.46, − 2.82	− 9.47	3.45	0.006	− 16.25, − 2.68
Body Mass Index	− 0.78	0.23	0.001	− 1.22, − 0.33	− 0.65	0.22	0.004	− 1.09, − 0.21	− 0.71	0.23	0.002	− 1.16, − 0.27
Sef-Efficacy					− 2.94	1.74	0.092	− 6.36, 0.48	− 3.22	1.73	0.064	− 6.63, 0.19
Autonomous Motivation					6.41	1.98	0.001	2.52, 10.30	6.91	1.98	0.001	3.02, 10.81
Controlled Motivation					− 1.17	1.84	0.527	− 4.80, 2.46	− 2.35	1.92	0.222	− 6.13, 1.43
Social Support from Family					0.68	1.8	0.708	− 2.87, 4.22	1.08	1.80	0.548	− 2.46, 4.63
SWS—Obligation to Help Others									3.90	1.88	0.039	0.20, 7.60

SWS = Superwoman schema; LPA = light physical activity

**Table 4:** Linear regression models predicting average daily minutes of MVPA (square root transformed) from demographics, social-motivational factors and SWS-obligation to help others

	Model 1				Model 2				Model 3			
	B	SE	*p*	95% CI	B	SE	*p*	95% CI	B	SE	*p*	95% CI
Intercept	4.80	0.13	< 0.001	4.53, 5.06	4.78	0.13	< 0.001	4.52, 5.04	4.78	0.13	< 0.001	4.52, 5.04
Age	− 0.02	0.01	< 0.001	− 0.04, − 0.01	− 0.03	0.01	< 0.001	− 0.04, − 0.02	− 0.03	0.01	< 0.001	− 0.04, − 0.02
Children at home	0.08	0.17	0.666	− 0.27, 0.42	0.04	0.17	0.800	− 0.30, 0.38	0.04	0.17	0.826	− 0.31, 0.38
Married	0.11	0.14	0.428	− 0.17, 0.39	0.13	0.14	0.341	− 0.14, 0.41	0.13	0.14	0.344	− 0.14, 0.41
Education	− 0.17	0.14	0.221	− 0.45, 0.10	− 0.13	0.14	0.341	− 0.41, 0.14	− 0.13	0.14	0.344	− 0.41, 0.14
Body Mass Index	− 0.02	0.01	0.090	− 0.03, 0.00	− 0.01	0.01	0.235	− 0.03, 0.01	− 0.01	0.01	0.230	− 0.03, 0.01
Self-Efficacy					− 0.01	0.07	0.916	− 0.15, 0.13	− 0.01	0.07	0.904	− 0.15, 0.13
Autonomous Motivation					0.26	0.08	0.001	0.11, 0.42	0.27	0.08	0.001	0.11, 0.42
Controlled Motivation					− 0.09	0.07	0.243	− 0.23, 0.06	− 0.09	0.08	0.242	− 0.25, 0.06
Social Support from Family					0.01	0.07	0.855	− 0.13, 0.16	0.01	0.07	0.839	− 0.13, 0.16
SWS—Obligation to Help Others									0.01	0.08	0.845	− 0.14, 0.17

SWS = Superwoman schema; MVPA = moderate to vigorous physical activity

### Follow-up analyses on self-determined motivation and the SWS-obligation to help others

Follow-up analyses were conducted predicting LPA and MVPA, with the final model including covariates (age, BMI, marital status, children living at home, and education), all the motivation sub-types, and the ‘obligation to help others’ subscale of the SWS in the final model. The final model for LPA was significant, *F*(11,289) = 4.76, *p* < 0.001, *R*^2^ = 0.15, and revealed significant positive main effects for intrinsic motivation (*B* = 7.96, *SE* = 3.16, *p* = 0.012) and the ‘obligation to help others’ subscale of the SWS (*B* = 3.82, *SE* = 1.85, *p* = 0.040). None of the other motivation types were significant. The final model for MVPA was also significant, *F*(11,289) = 5.50, *p* < 0.001, *R*^2^ = 0.17, and revealed a significant positive main effect for intrinsic motivation (*B* = 0.37, *SE* = 0.13, *p* = 0.003) and a significant negative main effect for identified motivation (*B* = − 0.25, *SE* = 0.11, *p* = 0.026). There were no significant effects for the ‘obligation to help others’ subscale of the SWS or the other motivation types.

## Discussion

The present study aimed to examine the relationship between the ‘obligation to help others’ subscale of the SWS and objectively-assessed LPA and MVPA among African American women, while also accounting for key social-motivational constructs (e.g., self-efficacy, social support from family, autonomous motivation, controlled motivation). Specifically, greater endorsement of the ‘obligation to help others’ subscale of the SWS was significantly associated with greater daily minutes of LPA, but not MVPA. Importantly, the effect of the ‘obligation to help others’ subscale of the SWS significantly accounted for unique variance in the prediction of LPA, but not MVPA. Among the social-motivational constructs, only autonomous motivation for PA was significantly associated with greater daily LPA and MVPA.

While past studies have found that greater endorsement of the SWS was associated with lower self-reported PA (Farr et al., 2025; Woods-Giscombe et al., 2019), to our knowledge, this is the first study on the SWS to include objectively-measured PA and to differentiate between PA intensities. Surprisingly, the present study found that greater endorsement of the ‘obligation to help others’ subscale of SWS was associated with higher daily LPA, which was contrary to our hypothesis. Drawing from past research which has found that caregiver responsibilities and lack of childcare are often perceived as barriers to PA among African American women (Joseph et al., 2015; Mathew Joseph et al., 2018; Obi et al., 2022; Siddiqi et al., 2011), it may be that the day-to-day activities of helping others over oneself (e.g., household responsibilities, chores, childcare) results in light physical activities that were not captured in past studies, especially given that self-reported measures of PA typically focus on MVPA. Given that LPA affords numerous health benefits, including improved cardio-metabolic health, weight status, healthier ageing, and reduced depression (Bourdier et al., 2023; Chastin et al., 2019; Hamm et al., 2025; Ku et al., 2018), future PA interventions for African American women may consider how to capitalize on engagement in light activities and pathways for transitioning from light to more moderate to vigorous activities. Importantly, past research has found that leisure-time PA is associated with greater health benefits than occupational PA (Gallagher & Carr, 2021; Holtermann et al., 2012; Janssen & Voelcker-Rehage, 2023), suggesting that it may be important in future research with African American women to evaluate whether LPA resulting from caregiving has the same health benefits as LPA arising from leisure-time activities.

Drawing from research on cultural tailoring, which emphasizes the importance of addressing ‘deep’ level structures (e.g., norms, values, beliefs) (Kreuter & Haughton, 2006; Resnicow et al., 1999), future PA interventions for African American women may benefit from a more targeted approach that addresses PA barriers and enhances sources of strength and resiliency related to the SWS, including culturally-relevant approaches to practicing self-care, accepting help, expressing strength and vulnerability, and managing stress. Furthermore, future interventions may benefit from strategies that aim to enhance autonomous motivation for PA within the context of the SWS. Efforts to increase autonomous motivation for health behavior change focus on enhancing competency, relatedness, and autonomy, with many studies drawing from motivational interviewing techniques, including eliciting change talk, using open-ended questions, and applying values exploration (Gillison et al., 2019; Resnicow et al., 2015). These approaches could be adapted to specifically address barriers related to the SWS (e.g., managing stress, expressing vulnerability) and find overlap between values related to the SWS and health (e.g., taking time for oneself to be a strong provider or role-model, expressing strength through PA). Past research also suggests that health disparities among African American (versus White) women (e.g., self-rated physical health and chronic conditions) are largely explained by socioeconomic status and racism-related stressors (Smith, 2021), highlighting the importance of considering multi-level intervention strategies that also consider broader structural determinants of health and societal pressures, such as caregiving expectations, economic strain, and limited access to childcare. Taken together, future interventions applying multi-level and culturally relevant approaches could help to offset the potential maladaptive correlates of the SWS on health as observed in prior research (Farr et al., 2025; Harris et al., 2022; Lewis et al., 2023; Mclaurin-Jones et al., 2021; Perez et al., 2023; Volpe et al., 2024; Woods-Giscombe et al., 2019), while also providing a pathway to optimize aspects of strength and resiliency encompassed within the SWS.

The present study found that among the social-motivational constructs only autonomous motivation was a significant predictor LPA and MVPA. Follow-up analyses revealed that among the different types of motivation, intrinsic motivation for PA had the strongest association with LPA and MVPA, which is consistent with past systematic reviews comparing the effects of different types of motivation (Rhodes et al. 2009; Teixeira et al. 2012a, b; Xu et al. 2025). Previous research evaluating the association between social support and PA among African American women has been mixed, including both positive associations and non-significant associations (Andersen et al., 2015; Gothe, 2018; Johnson et al., 2014; Sharma et al., 2005). One recent study found that the source of social support may matter, such that social support from family was more impactful on MVPA among older (versus middle-aged) African American women (Sweeney et al., 2026). Given the age range of the present study, this may help to explain the non-significant association between PA and social support from family. Furthermore, while self-efficacy is often considered a strong predictor of PA (Bauman et al., 2012; Choi et al., 2017), some studies have found that individuals initiating health behavior change are prone to overestimating their capability, as evidenced by decreases in self-efficacy over time (Barg et al., 2012; Buckley, 2016). Thus, future research may benefit from evaluating social support from multiple sources and the longitudinal associations between these social-motivational factors and the SWS among African American women who are initiating PA.

The present research also has implications for evaluating potential underlying pathways by which the SWS relates to health outcomes in African American women. Interestingly, the ‘obligation to help others’ component of the SWS was significantly associated with controlled motivation, but not with self-efficacy, social support from family, or autonomous motivation. Controlled motivation, which is characterized by feelings of guilt, obligation, and external pressure, relates to short-term PA, whereas autonomous motivation is more strongly associated with long-term PA (Fortier et al., 2012; Ryan et al., 2008; Teixeira et al., 2012a, b). Autonomous motivation is also more strongly associated with the long-term psychological health benefits associated with PA (e.g., quality of life, mental health) (Ntoumanis et al., 2020). Future longitudinal research is needed to evaluate whether the SWS is a relatively stable individual difference or whether changes in health behaviors are also accompanied by changes in endorsement of different aspects of the SWS and potential corresponding increases in autonomous motivation and decreases in controlled motivation. To the authors’ knowledge, no prior research has evaluated the relationship between the SWS and self-determined motivation, and all prior research on the SWS has been cross-sectional. Thus, future research is needed to evaluate the longitudinal pathways and underlying mechanisms by which the SWS relates to long-term health and well-being among African American women.

### Strengths and limitations

The present study has several strengths, including the use of an objective measure of PA, differentiating between PA intensities, a large sample size, and integration of both cultural and social-motivational theory-based constructs. The present study adds to a growing body of literature that aims to understand how the SWS and other socio-cultural factors relates to physical health outcomes among African American women (Farr et al., 2025; Harris et al., 2022; Lewis et al., 2023; Mclaurin-Jones et al., 2021; Perez et al., 2023; Volpe et al., 2024; Woods-Giscombe et al., 2019). While past research has focused on the bivariate association between the SWS and health behaviors (Farr et al., 2025; Woods-Giscombe et al., 2019), the present research is the first to our knowledge to demonstrate that the ‘obligation to help others’ component of the SWS is an independent and significant predictor of LPA when simultaneously accounting for key social-motivational factors, highlighting the importance of considering the SWS in the design of PA interventions for African American women.

However, there are also some limitations that should be acknowledged. The present study included baseline data from women who had recently enrolled in a PA intervention and were at least somewhat motivated to become more physically active, which may have impacted the results. Participants were eligible for the larger TEAM-PA trial if they engaged in less than 60 min of MVPA/week, which may have restricted the range for the PA outcomes, and impacted the non-significant results for MVPA. Individuals were excluded from enrolling in the larger TEAM-PA trial if they had a health condition that limited the ability to engage in PA, including uncontrolled hypertension, which may impact the generalizability of the findings. Overall, 53.8% of the study sample had hypertension at baseline, which is somewhat lower than prevalence estimates for African American women from national datasets (59.2%) (Palaniappan et al., 2026). Alternatively, 77.4% of the study had obesity, which is higher than prevalence estimates for African American women from national datasets (57.9%) (Palaniappan et al., 2026).

Due to efforts to minimize participant burden, the larger TEAM-PA trial only included one of the five subscales from the Giscombe Superwoman Schema Questionnaire, which did not allow us to draw comparisons across the different components of the SWS. The addition of the ‘obligation to help others’ component of the SWS significantly improved the model for LPA and yielded a significant main effect, but the overall effect size was relatively small. However, we propose that it is noteworthy that the ‘obligation to help others’ component of the SWS accounted for unique variance when simultaneously accounting for social-motivational constructs that have often been viewed as key mechanisms of change in past PA interventions (Rhodes et al., 2021). Relatedly, the larger TEAM-PA trial draws on components of Social Cognitive Theory and Self-Determination Theory, but we did not include measures for all components of these theories, which further limited our ability to fully evaluate and compare theoretical frameworks in the present study.

## Conclusion

This study highlights the importance of integrating both cultural and theoretical frameworks for understanding correlates of PA among African American women. Specifically, this study is the first to our knowledge to demonstrate that the ‘obligation to help others’ component of the SWS relates to greater LPA and has an independent association with LPA when simultaneously accounting for social-motivational factors. Taken together, these findings contribute to a growing area of research seeking to understand how the SWS and related social, cultural, and historical factors for understanding health and well-being among African American women (Farr et al., 2025; Harris et al., 2022; Lewis et al., 2023; Mclaurin-Jones et al., 2021; Perez et al., 2023; Volpe et al., 2024; Woods-Giscombé, 2010; Woods-Giscombe et al., 2019). Future research is needed to evaluate how barriers and facilitators related to the SWS can be addressed through cultural tailoring and multi-level strategies in PA interventions for African American women and how the SWS relates to changes in health outcomes over time.
